# Barriers and facilitators to providing rehabilitation for long-term care residents with dementia: a qualitative study

**DOI:** 10.1186/s12877-024-05433-z

**Published:** 2024-10-15

**Authors:** Michael Chigozie Ibekaku, Sara Ripley, Niousha Alizadehsaravi, Rebecca Affoo, Laura E. Middleton, Elaine Moody, Parisa Ghanouni, Lori E. Weeks, Caitlin McArthur

**Affiliations:** 1 School of Physiotherapy, Dalhousie University, Halifax, Canada; 2 School of Communication Sciences and Disorders, Dalhousie University, Halifax, Canada; 3 Department of Kinesiology and Health Sciences, University of Waterloo, Waterloo, Canada; 4 School of Health Administration, Dalhousie University, Halifax, Canada; 5 Aligning Health Needs With Evidence for Transformative Change (AH-NET-C): A JBI Centre of Excellence, Dalhousie University, Halifax, Canada; 6 School of Occupational Therapy, Dalhousie University, Halifax, Canada; 7 Schlegel-UW Research Institute for Aging, University of Waterloo, Waterloo, Canada

**Keywords:** Long-term care, Dementia, Rehabilitation, Barriers, Facilitators, Behaviour change, COM-B model, Canada

## Abstract

**Background:**

Rehabilitation can help long-term care (LTC) residents with dementia maintain their independence. However, many residents do not receive rehabilitation. This study aimed to identify the barriers and facilitators to providing rehabilitation for LTC residents with dementia and propose practical interventions for overcoming them.

**Methods:**

Using a phenomenological research design, we conducted a qualitative study involving 17 privately owned LTC homes in Nova Scotia, Canada. Data were collected through individual interviews and a focus group with residents with dementia (*n* = 3), family members (*n* = 4), rehabilitation providers (*n* = 6), and other staff (*n* = 3). We analyzed the data using inductive thematic content analysis and mapped the results onto the socioecological framework and the Behaviour Change Wheel (BCW) to classify and analyze barriers and facilitators to rehabilitation. The APEASE criteria (Acceptability, Practicability, Effectiveness, Affordability, Side-effects, and Equity) in the BCW were used to identify feasible interventions and policies linked to the identified barriers and facilitators.

**Results:**

Barriers at the intrapersonal level included communication difficulties, comorbidities, and lack of motivation among residents. Interpersonal factors encompassed the availability of family support and lack of interdisciplinary practice. Policy/environmental factors involved limited resources, complex admission processes, low staff ratios, and restrictive restraint policies. Enhancing communication, reducing the use of restraints, promoting interdisciplinary practice, and increasing accessibility to activity spaces and equipment will improve the provision of rehabilitation for the residents.

**Conclusion:**

Enhancing the capabilities, opportunities, and motivations of all actors in LTC homes can potentially minimize these barriers. Interventions such as staff training on effective communication and dementia care, promoting person-centred and meaningful activities, and improving interdisciplinary collaboration are crucial. Policy measures to improve hospital-to-LTC transitions, increase volunteer involvement, educate families and communities, and recruit more staff are recommended. Addressing these barriers through targeted interventions and policy changes can significantly improve rehabilitation provision for residents with dementia in LTC settings.

**Supplementary Information:**

The online version contains supplementary material available at 10.1186/s12877-024-05433-z.

## Introduction

Currently, it is estimated that there are 733,040 older adults in Canada living with dementia, and researchers are projecting that the number will increase to nearly a million in 2030 and 1.7 million by 2050 [1]. Many people with dementia will require long-term care (LTC), defined as residential care for individuals who are unable to maintain independent living and require ongoing nursing, personal, and supportive care [2]. The Canadian Institute for Health Information estimated that one-third of older adults younger than 80 years who have been diagnosed with dementia are living in LTC with the proportion increasing to 42% for those aged above 80 years [3].

LTC residents with dementia often experience high levels of functional impairment and disability leading to reduced quality of life [4–7]. Most require support to perform activities of daily living (ADL) [8]. ADLs are a range of fundamental skills required for safe and independent living and include eating, bathing, and mobility [9]. The inability to perform these tasks is an indicator of poor functional health status resulting in dependence on other individuals to maintain a living that is consistent with basic human dignity [10].

Physical function and quality of life of people with dementia can be improved through physical rehabilitation. Defined as interventions for people experiencing or at risk of experiencing functional limitation, physical rehabilitation includes activities such as strength and functional training, walking, and balance exercises [11]. For example, a clinical trial showed that LTC residents with dementia who participated in a 4-month walking intervention experienced an improvement in health outcomes such as the Alzheimer's Disease-related quality of life, Functional Independence Measure, Timed Up and Go, and the 2-min walk test [12]. However, many LTC residents in Canada do not receive any form of rehabilitation [13]. Indeed, only 22 to 55% of LTC residents receive any physiotherapy or occupational therapy [8, 13]. Even more concerning is that residents with dementia are less likely to receive rehabilitation interventions than those without [13]. The disparity in the proportion of LTC residents requiring and receiving rehabilitation presents an opportunity to improve equitable healthcare provision for this population through rehabilitation.

The identification of factors limiting the provision of rehabilitation services for LTC residents with dementia is an important first step toward addressing the shortfall in rehabilitation provision. Previous studies identified poor outcomes of interventions, and a lack of training and educational resources as limiting factors in the provision of rehabilitation for people with dementia in the community [14–16]. This suggests that providing more training and resources for providers might improve rehabilitation provision for people with dementia. Moreover, it was also reported that healthcare professionals’ knowledge of dementia, positive personal values, and cultural awareness can improve physical rehabilitation interventions for LTC residents [17]. However, factors limiting the provision of rehabilitation for older adults living with dementia in LTC homes in Canada have not been reported. Therefore, an examination of the barriers and facilitators to providing rehabilitation for LTC residents with dementia is necessary.

Once these factors are identified, they could be used to advocate for changes in practice which could result in more equitable access to rehabilitation in LTC. The Behaviour Change Wheel (BCW) is a model that employs behaviour change theory to guide the comprehension, selection, and specification of the target behaviour before creating interventions [18]. The capability, opportunity, and motivation behaviour model (COM-B) is an integral component of the BCW which helps to identify which capabilities, opportunities, and motivation are barriers to the execution of a desired behaviour [18]. Once these are identified, they are then linked to intervention functions and policies to drive the desired change. The APEASE criteria (Acceptability, Practicability, Effectiveness, Affordability, Side-effects, and Equity) is used to determine the feasibility of implementing the identified intervention functions and policies [18]. Additionally, the socioecological model (SEM) breaks down barriers and facilitators in a system, such as the healthcare system, by examining factors at multiple levels: intrapersonal, interpersonal, and institutional/public policy. This comprehensive approach helps identify and address the complex interactions between different levels that influence behaviour and outcomes. For instance, in healthcare, it can reveal how individual attitudes, social networks, organizational policies, community relationships, and governmental regulations collectively impact healthcare access and utilization [19].

Using the BCW and the SEM as the guiding frameworks, we aimed (1) to identify barriers and facilitators to providing physical rehabilitation for LTC residents with dementia from the perspectives of residents, family members, and LTC staff and (2) to identify interventions and policies linked to the barriers and facilitators using the COM-B model.

## Methods

### Study design

A phenomenological research design [20] was used to explore LTC residents, their family members, nursing staff, and rehabilitation providers’ perceived facilitators and barriers to rehabilitation for LTC residents with dementia. We considered phenomenology as the most appropriate approach in this research as it allowed us to explore our research questions through the participants’ lived experiences [21]. We collected qualitative data through individual in-depth interviews and a focus group with rehabilitation providers working in LTC (physiotherapists, occupational therapists, and physiotherapy/occupational therapy assistants), nursing staff, family members, and residents living with dementia. The study outcomes were reported according to the consolidated criteria for the reporting of qualitative research [22] (see Additional file 1).

#### Setting

This study was conducted across 17 privately owned LTC homes in Nova Scotia, Canada. Participants were recruited by the primary investigator and a research assistant over 6 months between October 2022 and May 2023. Each LTC home received an email and a flyer with information about the study to distribute to staff, family members, and residents.

#### Selection criteria

We included four population groups to gain a holistic perspective of rehabilitation in LTC. These include (a) rehabilitation providers, (b) residents with dementia, (c) family members of residents with dementia, and (d) LTC staff who provide care for residents with dementia. Eligible rehabilitation providers were physiotherapists, physiotherapy assistants, occupational therapists, and occupational therapy assistants who had worked in LTC for a minimum of six months. LTC residents were eligible to participate if they had a diagnosis of dementia (any type), could communicate with the interviewer (e.g., able to speak, write, draw), and had been living in LTC for at least 2 months. Residents receiving palliative or end of life care were excluded from the study. Family members were eligible to participate if they were associated with a LTC resident diagnosed with dementia who was not receiving palliative or end of life care. Finally, LTC staff providing direct care to residents with dementia (e.g., health care aides, nurses) who had worked in LTC for six months or greater were eligible for inclusion in the study. The inability to communicate in English was a general exclusion criterion across all participant groups. We aimed to recruit 5–6 participants per group as previous studies suggest that this will be sufficient to understand the phenomenon studied [23]. We confirmed data saturation during the coding and analysis phase when similar themes repeatedly emerged after analyzing the fourth and fifth transcripts of each group.

#### Participants selection

LTC staff (registered nurses, licensed practical nurses, administrators, physiotherapists, and occupational therapists) within each LTC home identified residents, family members, and other staff members who may be interested in participating and provided them with the study information flyer. Interested participants contacted the research assistant who provided them with more information about the study and the research team and determined their eligibility. To recruit LTC residents, information was provided to the residents and their substitute decision-makers. Interested residents and substitute decision makers contacted the research assistant and the same process as described above occurred.

#### Data collection

Data were collected through individual interviews and a focus group over the period of 3 months based on participants' preferences and convenience. We completed individual interviews with residents living with dementia (*n* = 4), rehabilitation providers (*n* = 6), registered nurses (*n* = 3), and family members (*n* = 4). The focus group included three nursing staff and one family member. The individual interviews lasted an average of 30 min while the focus group lasted for 60 min. We did not record any dropouts as all participants who indicated interest completed the study.

Most of the interviews including the focus group were conducted in-person in a quiet space (e.g., a meeting room) by the principal investigator with only the participant present. However, three of the interviews were conducted virtually via Microsoft Teams where the participants preferred to participate online. Before the interview, participants were asked demographic questions (e.g., age, gender, time worked or lived in LTC) by the researchers. The interviews and the focus group were guided by a semi-structured guide developed in collaboration with our project advisory committee to answer the identified research questions (see Additional file 2). This committee comprised researchers, clinicians, and patient and public partners with lived experience with dementia and the LTC setting. The interviews and the focus group were audio recorded and the principal investigator took notes during each interview and applied them as prompts in subsequent interviews. The audio was automatically transcribed using Otter.ai [24] and the transcripts generated from Otter.ai were reviewed for correctness by a research assistant.

### Analysis and findings

#### Identifying themes

Data was analyzed using the principles of thematic content analysis [25]. Inductive thematic content analysis allows for systematic identification and analysis of recurring themes, providing a structured yet nuanced data analysis [26, 27]. Two coders independently identified and defined the codes or units of analysis which are words, sentences, or groups of words describing the barriers and facilitators. They met three times to discuss and reconcile discrepancies in their analysis. This level of analysis was completed in Nvivo 12.

#### Classification of themes into levels of the socioecological model

**Fig. 1:**
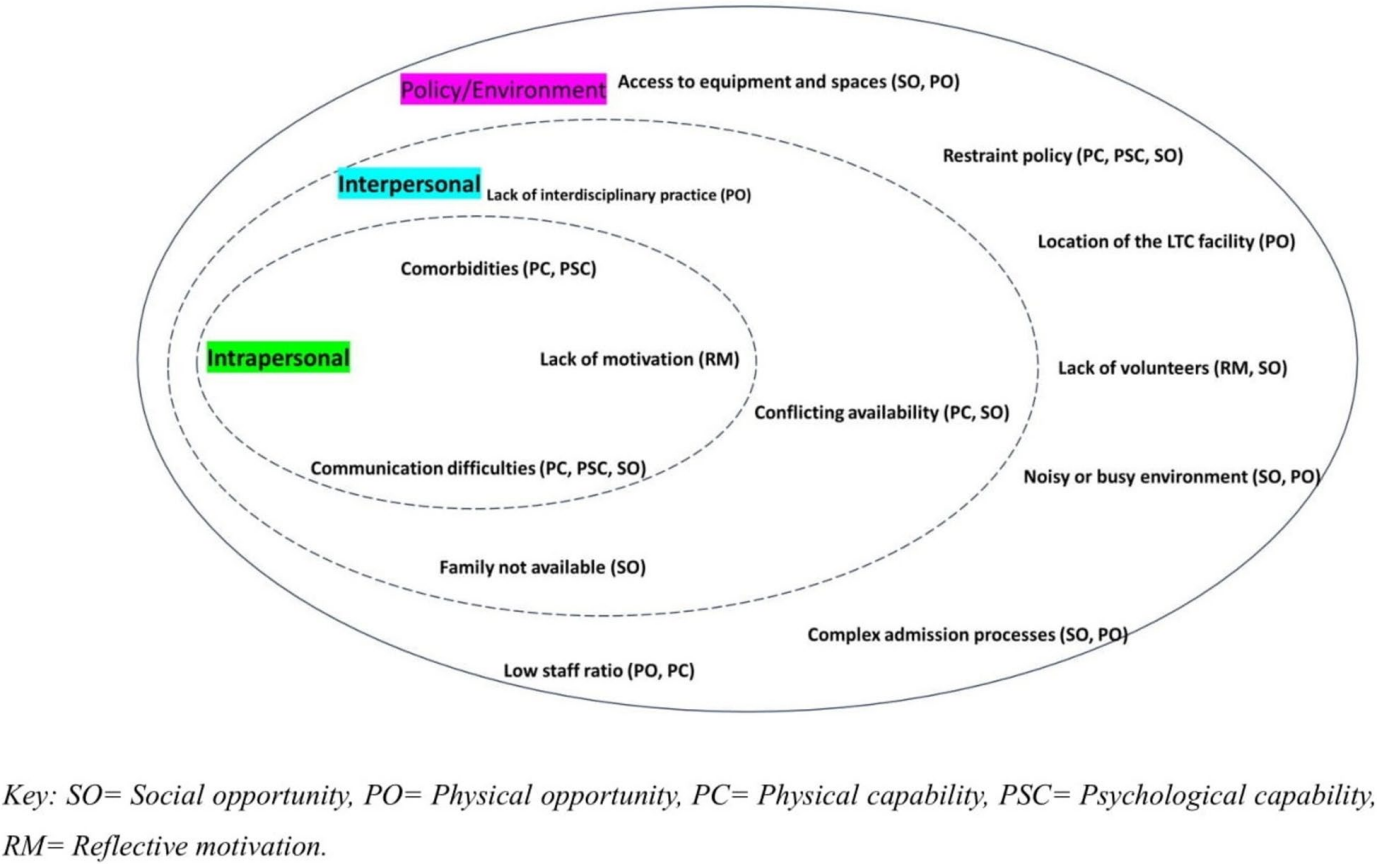
Classification of themes into socioecological framework

Using the SEM [28], we classified the codes/themes into intrapersonal, interpersonal, and policy/environmental factors affecting the provision of rehabilitation for LTC residents with dementia (see Fig. 1). Intrapersonal factors involve individual characteristics such as knowledge, attitudes, beliefs, and skills. For example, a resident's motivation and physical capability to participate in rehabilitation activities were considered intrapersonal factors. Interpersonal factors encompass relationships and social networks, such as support from family, friends, caregivers, and healthcare providers, which can influence a resident's engagement in rehabilitation, while policy/environmental factors include broader organizational, community, and policy influences. For example, healthcare policies, the physical environment of the LTC facility, and the availability of rehabilitation resources and services were considered policy/environmental factors.

#### Mapping onto COM-B model

The behaviour of interest is the provision of rehabilitation for LTC residents with dementia. These services include physical activities and exercises that improve their health and quality of life. Using COM-B, the barriers and facilitators were identified as capabilities (physical and psychological), opportunities (social and physical), or motivations (reflective and automatic). Capability is defined as an individual’s physical or psychological ability to execute the desired behaviour. Opportunities are social and physical factors that lie outside of the individual that can facilitate the desired change while motivation is the cognitive factors that stimulate and direct behavioural change [18]. After completing the coding and thematic analysis, we identified what needs to change to improve the provision of rehabilitation for residents and suggested ways of achieving the desired change by linking the COM-B to intervention functions and policy categories (see Table 1). Then using the APEASE criteria in the BCW framework, we analyzed the plausibility of implementing the linked intervention functions and policy categories (see Table 2). Only the intervention functions and policy categories that met the APEASE criteria are discussed in the results. This step was completed independently by three of the team members including the two who completed the initial coding. Differences were discussed and reconciled in a series of three meetings with the rest of the team.

**Table 1:** Classification of identified themes using the socioecological framework and the COM-B Model

Level of analysis	Themes	COM-B theme	Summary quote	Full quote	Desired change
Policy/environmental factors	Access to equipment and spaces	Social opportunity Physical opportunity	*We don't have the equipment...* *They only have two machines I can use...* *I haven't been able to do since I've been here...* *You don't have the financial means...* *There’s no space where we can use...*	*I'm submitting something for you today to get you a wheelchair. It might not show up for the next six months. So what do we do with you in those six months? If you need it in the next six weeks while we’re trying to rehab you, we don't have a chair for you to go in, you're spending all that time in bed, even just to sit up and foot propel or mobilize or just have that hip bent at 90 degrees is going to benefit you. So if we don't have the equipment to appropriately do that, it just kind of puts us behind…..Rehabilitation Provider7* *I go there but they only have two machines I can use okay. Yeah. So again, it limits. One of my personal goals is to lose weight. But, is very slow going there, you're so limited ….Resident 2* *I prefer to take a walk around the block, which I haven't been able to do since I've been here this year….Resident 3* *So all of these preventative measures to stop the need from the rehabilitation, which you know, we want to be proactive about it. There's just a big barrier there as well. Just because if people don't have it or you don't have family involved or you don't have the financial means to do it, then you just don't get it….Rehabilitation Provider8* *there's no dedicated rehab space or, you know, space where we can use to bring somebody away from those distractions into an area where they can actually focus on what they're doing. So, I find that's a big challenge across all the homes. Umm, I work across four homes, um, and the setup is pretty similar in each of them…….. I would love, for example, a mechanical, a walking harness type system where you know, we can practice those, balance things, where they’re actually hooked up to something and they're not gonna fall. That's my biggest fear when we're trying to really, like, stretch the boundaries of that is having a safe area to be able to do that in….Rehabilitation Provider5*	Improve access to equipment and create rehabilitation spaces
	Complex admission processes	Social opportunity Physical opportunity	*Getting that piece that takes up a lot of our time...* *Their services in the hospital are really scaled back...* *We're not receiving accurate reports from the hospital...*	*So it's and getting that piece that takes up a lot of our time. Working on those admissions and trying to get equipment and trying to get, you know, things in place for them to even be successful on admission and to have, it's important to have that success within those first few days because you know their first impression is everything and they're, um, if they become agitated and anxious because we haven't got things in place, then we're yeah, it's difficult to come back from that…Rehabilitation Provider5* *I find in hospital their services in the hospital are really scaled back because they're waiting for long term care and obviously the rehab departments in the hospital have all these other people that possibly are going home that they then focus their time on, which is a shame, because if they, if there was more focus then on those people coming to long term care by the time they get to us they would be in a better physical condition to be able to actually participate when they get here, but often we find they have spent many, many weeks in a Broda chair, restrained and medicated…..Rehabilitation Provider5* *We're not receiving accurate reports from NSHA when we have somebody coming back, you know, we get discharge papers, they're spotty, they say. Ohh, mobilize this, this times two with physiotherapy, but then we get reports from the family that physiotherapy was never there and they never got out of bed. And then OK, so like, they're saying an assist times two at the hospital, they come back, they can't stand up and we're using mechanical lift and we're trying to rehab from that. that's definitely a barrier rehabilitation because they say, OK, well, they said at the hospital they should be doing this or they were able to do this and now they're not able to. So why can't they do it with you guys?…Rehabilitation Provider7*	Streamline admission process Improve hospital to LTC transition process
	High resident turnover and low staff ratio	Physical opportunity Physical capability	*Sometimes I was the only nurse...* *But I’m lucky if I get the one,...* *We have to write those notes...* *You have to start picking and choosing...*	*I had 58 demented residents to look after. And I'm not talking early dementia. I'm talking about very advanced dementia that nobody else looked after because there was nowhere else in the building and sometimes I was the only nurse….Staff2* *We only have one physio, one occupational therapist, right and two assistants, for the whole building, for like, 300 residents……. The previous home no one saw me. But here, officially I'm supposed to get three visits a week. But I’m lucky if I get the one…..Resident 2* *And then we have to write notes like chart notes and progress notes. So if we don't have to write those, then we have extra time to see more people. But then we have to write those so. Yeah, that makes sense too, yeah….Rehabilitation Provider8* *So then it's like, what's priority, who do we have to see? Who's gonna get the max benefit from what we're offering? Because then you have to start picking and choosing like who you're going to see and why you're gonna see them and what's the benefit…..Rehabilitation Provider7*	Recruit more care providers
	Lack of volunteers due to COVID-19	Social opportunity Reflective motivation	*People do not want to come in here...*	*I still think COVID is a big issue. People do not want to come in here. They think they're gonna catch something or they're gonna bring it. Yeah. I think that's still the issue, but and the barriers to volunteerism, like, I just don't know why we haven't moved forward….Staff1*	Attract more volunteers
	Location of the LTC facility	Physical opportunity	*Where the homes are situated are often not ideal...* *I feel safer up there than here...*	*This home here is in [location] and is at the top of a steep hill so you know we could go out onto the, but they'd be very quickly at the bottom so. It's yeah, the where the homes are situated are often not ideal. I often feel jealous when I see places like [LTC home] and that that are like right in the center where you could go and go to Tim’s or, you know, do a walk to the store or something like that would be would be nice. But yeah, we have very few like functional active outdoor activities where we could take them…Rehabilitation Provider5* *I did live in [location]. Okay, and then my husband and I moved to [location]. There’s good walking up there. In fact, I feel safer up there than I do in [location]….Resident 2*	Site facilities in good locations
	Restraint policy	Physical and psychological capability Social opportunity	*They're going to fall...* *So they're not tearing at the chair...*	*we're very risk-averse within long-term care. So I find that nursing staff don't want people falling. Like if somebody has a fall, we go home for the weekend, we come back and they're restrained in a wheelchair. And they've had that conversation with the families. And they said, you know, they're gonna, they're gonna fall. They're gonna break something. We need to have them restrained in this wheelchair for their safety Coming back from that is very difficult from a personal perspective, because if I'm then going to the families, do you know what? You know, quality of life versus quantity versus and you have that conversation and you they agree to then take the restraint away and then they fall and they break a hip. Then all of a sudden, you know the physio told me that…Rehabilitation Provider5* *We give them medications to settle them down. So they're not tearing at the chair and trying to get him so, it's a balance….Staff2*	Reconsider mobility needs of residents
Interpersonal factors	Family not available	Social opportunity	*They provide tips and tricks...* *That can be a big benefit if you have somebody...* *Primary caregiver must be present...* *My loved one is not being restored to where they should be...*	*Family can be helpful sometimes if you can get a family member, a loved one to call and encourage them to participate in rehab….Rehabilitation Provider1* *PT might put out some information for families, you know, basic range of motion if they're looking for something that they can do with mom or dad while they're visiting. Umm that can be a big benefit if you have somebody in there routinely that does it, but if you don't have a consistent family member, somebody willing to do that, that can kind of inhibit your recovery after an injury as well….Rehabilitation Provider7* *I believe that the government should have a requirement that anyone who is listed as the primary caregiver must be present at the facility at least twice a month….Family member 1* *there is an unrealistic expectation of care and almost an anger that my loved one is not being restored to where they should be. And I talked to half a dozen people that have a new person in and it's either about meds or this or that but they are also talking to that loved one….Family member 1*	Increase involvement of family support
	Noisy or busy environment	Social opportunity Physical opportunity	*There's so much holiday stuff going on...*	*And then there are some people I've been told basically won't leave their room for the next three weeks because there's so much holiday stuff going on. Umm, you know, I'm gonna try to hide some Christmas trees in closets and get people out anyway. But there's just so many different environmental factors that play into it, but a big one I find is like stimulation, so it's like. What's visually happening? What's happening? Auditory wise? Umm, who's around? I mean, my biggest facility has 60 people and if you put any human with 59 other humans, there's gonna be people they don't get along with or they just don't like….Rehabilitation Provider1*	Make the environment conducive
	Interdisciplinary practice	Physical opportunity	*Communication is really kind of important...*	*I think it makes it easier if, you know, you've got kind of sort of meeting such a risk meeting so we can actually discuss specific residents' needs and what interventions are needed. Obviously, we can't. We haven't got the same sort of communication between yourself and the resident. So you're relying very much on those around to actually glean the information So I think communication is really kind of important…Rehabilitation Provider3*	Adopt interdisciplinary practice
Intrapersonal factors	Communication difficulties	Physical capability Psychological capability Social opportunity	*They don't understand that we're trying to help...* *Communicate clearly, concisely, and loudly...*	*they're not overly understanding of why we're there or what the purpose of doing all of this is, they just feel the pain and they see us as the people that are coming to hurt them, essentially because they don't understand that we're trying to help them recover and get them back up on their feet, so that lack of understanding, difficulty with following directions, the compliance with going along with the program is a big barrier….Rehabilitation Provider7* *being able to communicate clearly, concisely, and loudly, sometimes with pictures, sometimes with tools. Again like bringing the walker to somebody. If they've used a walker, then they're gonna know what they're doing to a certain degree. So that can be really helpful….Rehabilitation Provider1*	Improve communication strategies
	Comorbidities	Physical capability Psychological capability	*If I feel annoyed to stretch i then I’ll stop...* *I get freaked out. Yeah. So I stay in my room mostly...* *It just doesn’t come back...* *Mental health piece is often seen as less of a priority...* *They have fear and they are like no, I cannot walk* *A lot of people are afraid to go down...* *It is so important to do the GPA training...*	*If I feel annoyed to stretch it [shoulder] then I’ll stop….. It’s [Arm circle movement] is harder [with a shirt on] and I also had this [shoulder problem] to cope with…Resident 1* *With my condition, when there's more than one or two around me, I get freaked out. Yeah. So I stay in my room mostly* *It can be as simple as a urinary tract infection that is that difference. So it's not just’ fractured hip or a broken arm or anything. It can be as simple as delirium. That has set in it and then, oh, my goodness, it’s just another, it just doesn’t come back…..Staff 1* *once somebody gets diagnosed with dementia, that mental health piece is often seen as less of a priority because of the they have dementia, so they the shift of focus changes into, OK, they have dementia, they have this progressive disorder. However, they have lived with a mental health disorder for many, many, many years, and now we’re not really dealing with that piece…Rehabilitation Provider5* *They have ability, I know, but they have fear and they are like no, I cannot walk. I cannot. They will do all kinds of exercises, I feel sometimes they have more strength than me. But then they won't stand up. They will do all kinds of exercises in bed or in chair, but they won't stand….Rehabilitation Provider8* *there’s a big hill at the entrance here. And a lot of people are afraid to go down, even in their chairs even with a seatbelt. I do it without the belt. I don't feel scared to do it. Yeah, but lot of people are…Resident 3* *So I think that it is so important to do the GPA training. Because you can see strategies you can understand more about this condition and how you can do to improve your. Your treatment, your. Your time with this person….Rehabilitation Provider2*	Assess and manage other health problems Staff should undergo GPA training
	Conflicting availability between residents and rehabilitation providers	Physical capability Social opportunity	*They're sleeping and there is no way I can get them up...* *We have an option to try another time...*	*A few residents sleep during the day and are up during the night, and I don't work during the night, so. It's so hard to get them to walk. They do have physical ability because during the night they are walking around the unit. But then the balance is not great and when I want them to walk, they're sleeping and there is no way I can get them up…..Rehabilitation Provider8* *If I try during the morning and I think that is not possible to do the exercise at this time here working at a long term care. We have an option to try another time like in the afternoon and maybe the afternoon there is it it's better….Rehabilitation Provider2*	Adopt flexible schedule
	Lack of motivation	Reflective motivation	*She's happy Waiting And sitting...* *The end destination is going to be the dining room...* *We start to talk about something that they like...* *They might go for a walk to see the bird feeders...*	*But she moved in the long into assisted living. She wasn't much interested in doing things unless they were just going somewhere and sitting, you know? But, you know, she wouldn't play like, a card game or bingo. No way. She'll be around people doing it. It's never been her thing anyway. Doesn't interest her. She's happy. Waiting. And sitting….Family member 1* *I've been doing that all my life, you know…Resident 1* *If somebody is food motivated, you know it's pretty easy to get them up for a walk if it's right before lunchtime because the last you know the end destination is going to be the dining room…. Rehabilitation Provider1* *sometimes we start to talk about something that they like or talk about the family or don't talk about the family. Depends on. Yeah. If this you they you be sad talking about some person they don't. We don't do that. It's important to know more details about the resident before you start doing the treatment…..Rehabilitation Provider2* *if you've got someone who's 80 and they haven't exercised intentionally, like, maybe they like hikes or what have you. But like, they would never were like I'm doing a leg workout today. They're not gonna do it now. They did 80 years without it. It's just not gonna happen. But they might go for a walk to see the bird feeders on the other side of the facility. Because they do like to see the birds and perfect. Well, you just got your leg workout in for them and they got to see the birds and. It's just different, different goals, the same activity…..Rehabilitation Provider1*	Adopt functional activities

**Table 2:** Mapping of themes onto BCW, using COM-B, Intervention functions, APEASE criteria and policy categories

**COM-B component (Barriers)**	**Is there a need for change?**	**Linked intervention functions**	**Does the intervention function meet the APEASE criteria?**	**Linked policy categories **
Social opportunity (Complex admission processes)	Yes: 1. Streamline admission process	Restriction	Yes: review and update regulations and procedures for admission into LTC either from home or acute care	Regulation: Set clear rules and regulations to guide hospitals to LTC transition.
(Low staff ratio)	2. Attract more volunteers	Enablement	Yes: create policies to encourage volunteers	Communication/marketing: Use mass media campaigns to sensitize people
(Restraint policy)	3. Reconsider the mobility needs of residents	Training	Yes: Train staff on how to effectively respond to responsive behaviours with minimal use of restraints	Guidelines: Create a policy guideline on improving mobility with minimal fall risk.
(Family not available)	4. Increase involvement of family support in care	Education	Yes: Educate patients about dementia and patient-centred care	Communication/marketing: Provide regular education sessions for family
(Noisy or busy environment)	5. Design conducive environments	Environmental restructuring	No: Not practicable as the environment accommodates several people with varying needs.	
Physical opportunity (Access to resources)	Yes: 1. Improve access to equipment and create rehabilitation spaces	Environmental restructuring	Yes: create a space for a gym or exercise room	Environmental/social planning, Fiscal measures
(Low staff ratio)	2. Recruit more care providers	Enablement	Yes: Employ more healthcare personnel to work in LTC	Fiscal Measure: Set rules on the minimum number of rehabilitation providers in LTC home
(Location of the LTC facility)	3. Build facilities in good locations	Environmental restructuring	No: Not affordable, not practicable	
(Lack of interdisciplinary practice)	4. Adopt interdisciplinary practice	Training	Yes: Improve interprofessional collaboration and communication	Regulation: Adopt interdisciplinary practice as the standard of practice.
Physical capability (Low staff ratio)	1. Adopt a flexible schedule	Persuasion	No: It might not be practicable due to excessive workload	-
(Communication difficulties)	2. Improve communication strategies	Modelling	Yes, use pictures and illustrations to demonstrate desired activity	Communication/marketing: with residents with dementia
Psychological capability (communication difficulties)	Yes: 1. Reduce agitation and fear of fall among residents	Education	Yes: members can be educated on the management of responsive behaviour	Communication/marketing: Educate providers on how to manage responsive behaviours
(communication difficulties)	2. Improve residents' communication capacity and the ability of providers to communicate with residents	Training	Yes: providers can be educated on appropriate communication strategies	Guidelines: Develop guidelines for engagement of patients living with dementia
Reflective and automatic motivation (Lack of motivation)	Yes: 1. Motivate residents to engage in rehabilitation activities	Persuasion	Yes: Adopt person centered care	Communication/marketing: Encourage adoption of person-centred care
(Lack of volunteers)	2. Attract more volunteers	Education	Yes: Promote volunteer opportunities in LTC	Communication/Marketing: Use mass media campaigns to sensitize people

### Ethical considerations

Ethical approval for this study was obtained from the Dalhousie University Health Sciences Research Ethics Board. All participants signed the informed consent form before participating in the study. The residents retained the autonomy over their decision to participate even if they had a substitute decision maker in place. In which case, the substitute decision maker provided written assent to participate in the study by signing the informed consent form. We maintained the anonymity of participants by replacing their personal identifiers with alphanumeric letters and removing names and other identifiers from the transcripts prior to analysis. We also ensured data privacy by storing data in encrypted devices.

### Research team and reflexivity

The principal investigator is an assistant professor with a background in physiotherapy. She is an experienced qualitative researcher with interests in rehabilitation for people with chronic health conditions across the continuum of care. All interviews were conducted by the principal investigator, while the primary coding and analysis tasks were carried out by a female research assistant specializing in audiology and a male PhD student with a background in physiotherapy. We ensured the trustworthiness of data through consistent peer debriefing throughout the data collection and analysis phases. All coding and mapping onto the COM-B were reviewed and confirmed by other team members who did not originally participate in the coding or mapping through peer debriefing led by the primary investigator.

## Reporting of findings

### Summary of participants’ characteristics

All four residents included in the study were females and their ages ranged from 65 to 85 years. They had been living with dementia for between 4 and 8 years and had been residents in LTC for an average of 2.5 years. The years of experience of working in LTC ranged from 11 to 29 years and 1.5 to 8 years for the staff and rehabilitation providers, respectively. We had only two males across all participant groups. While the data was analyzed by participant groups, the themes had contributions from all the groups.

### Barriers and facilitators to rehabilitation in LTC

The identified barriers to and facilitators of rehabilitation in LTC for people living with dementia in LTC are summarized using the socioecological framework and COM-B (see Fig. 1). See Table 1 for a summary of the themes and supporting quotations. All participant groups (i.e., residents, family members, staff, and rehabilitation providers) contributed to each theme.

### Intrapersonal level factors

#### Communication difficulties (physical and psychological capability, social opportunity)

Difficulties with communication were identified as a major barrier to the engagement of residents in rehabilitation activities. The rehabilitation providers indicated that as residents gradually lose the capacity to express their needs and to understand instructions, they might also struggle to understand the purpose of the activity or how to carry it out. For instance, some of the rehabilitation providers stated that some residents might think that the intention of an exercise was to hurt them and may become aggressive or agitated. As a result of the challenges with communication, our participants suggested ways of improving its effectiveness. They indicated that the communication strategies should be clear, concise, loud, and illustrated with pictures, gestures, or supported by assistive tools (e.g., walker). Our participants recommended the Gentle Persuasive Approach [29] as an important training for care providers working with people living with dementia, which emphasizes encouragement and functional activities to improve care providers' understanding of dementia and strategies for effective engagement of residents.

#### Comorbidities (physical and psychological capability)

Another barrier to rehabilitation was the onset of other health conditions. Residents stated that health conditions like joint limitations, anxiety prevent them from engaging in activities they would have normally participated in. Similarly, the staff indicated that the residents experience a steep decline in functioning and capacity to engage in activities when they have other morbidities like fractures, pain, or delirium. They revealed that residents typically do not recover their premorbid status after the onset of dementia. The coexistence of mental health problems with dementia also presents additional challenges for rehabilitation providers as mental health experts are not usually involved in care. Thus, the mental health of residents is often treated with less priority once they are diagnosed with dementia. The fear of falling was also identified as a barrier to rehabilitation. Some of the residents have the potential to mobilize and engage in activities but are not doing so because they are afraid of falling. Consequently, their exercises are limited to bed activities.

#### Lack of motivation (reflective motivation)

One of the challenges rehabilitation providers encounters is an apparent lack of motivation from residents to participate in any meaningful activities. It appears that some would not respond to attempts to get them involved and engaged. The residents indicated that concerns about safety and having too many people in close proximity discourage them from participating in activities, especially group activities. However, some of the residents expressed that they are motivated to engage in rehabilitation activities because they have been active all their lives. Family members reinforced that residents’ predisposition to engage in activities is consistent with their personality.

The rehabilitation providers and staff narrated that they attempt several strategies to motivate residents including identifying their sources of motivation, bringing their family or spouses during activity (when feasible), or engaging them in discussions about their life experiences. They noted that while these strategies are not guaranteed to work all the time, they can be useful in getting the resident to participate in some activities.

The rehabilitation providers indicated that the use of functional activities like playing cribbage, baking, or walking for sightseeing improves compliance with rehabilitation activities. Residents, especially those who are not used to structured exercises, can relate better to meaningful functional activities. In this case, instead of asking the residents to “exercise,” they might ask them to engage in activities they will normally perform like dancing or exploring the environment.

### Interpersonal-level factors

#### Family not available (social opportunity)

Our participants considered the involvement of relatives and family members as an important facilitator of the provision of rehabilitation for residents. The residents narrated how they used to engage in activities like walking with their loved ones before moving into the LTC home, they reported doing less walking presently. Rehabilitation providers also indicated that families provide “tips and tricks” for the engagement of residents in activities. In some cases, the Physiotherapists and Occupational Therapists instruct family members how to safely provide rehabilitation activities like range of motion exercises and walking for the resident.

On the other hand, some families expressed that their expectations about how their loved ones should be cared for were not being met. Some believe that the staff in LTC are not providing enough care for their loved ones, leading to frustration and anger. They often feel that the declining function of the residents is due to the poor quality of care they receive from the providers. Some advocated that there should be a form of education for families about dementia and how they can contribute to the care provision for the residents.

#### Lack of interdisciplinary practice (physical opportunity)

Our participants believed that collaborative effort among all caring professionals facilitates and improves the quality of care provision for residents. They highlighted the importance of interdisciplinary practices like communication, care, risk meetings, and coordinated care plans. However, it was noted that these practices even though desirable, are not commonly practiced due to time constraints and the heavy workload of the professionals.

#### Conflicting availability between residents and rehabilitation providers (physical capability, social opportunity)

The rehabilitation providers stated that one of the barriers to their work is having residents sleeping most times of the day, making it difficult to find a window of opportunity to work with them. It was indicated that for some residents, the wake cycle is reversed, where they are awake at night and sleeping during the day. The rehabilitation providers believe that the use of antipsychotic medication often contributes to extended sleep hours during the daytime. In response, some of the providers have adopted a flexible approach to their work, which includes varying the time they attend to the residents or varying their care plans. They also wanted the medications to be given at different times so that the residents could be more active during the day.

### Policy/environmental level factors

#### Access to resources (financial, equipment and space) (social and physical opportunity)

Equipment like mobility aids (e.g., wheelchairs), transfer belts, and standing frames would aid rehabilitation activities but are often not available in the homes resulting in frustrations for the rehabilitation providers and the patients. Similarly, rehabilitation activities are also limited by the lack of dedicated safe spaces like exercise rooms, or gyms. For instance, some residents stated that they would like to walk more frequently and visit the exercise room but are limited by safe spaces and appropriate equipment. Rehabilitation providers also highlighted that such equipment and spaces are important for safety, reduced distraction, and good interaction during rehabilitation activities. However, low access to these equipment and spaces is mostly due to financial limitations as most are expensive and with limited sources of funding.

#### Complex admission processes (social and physical opportunity)

The process of admitting residents into LTC homes was identified as a limiting factor in the planning and delivery of rehabilitation in the homes. Most of the residents are admitted at a very late stage of dementia when they have lost a considerable level of function, and late admission means that providers have a shorter time to plan and deliver quality care. In addition to late admission, it also takes some time to complete the paperwork and put resources in place which might affect the initiation of rehabilitation activities.

Transitions from acute care settings like hospitals to LTC are also complex and complicate the rehabilitation process. The respondents stated that when hospital patients are earmarked for admission to LTC, care priorities shift to those who are transitioning home, resulting in neglect of the hospital patients transitioning to LTC. In addition, these hospital patients experience prolonged wait times before eventual admission which often results in a decline in function when they get to LTC. There is also an apparent communication gap between the acute hospitals and the LTC homes where the discharge note does not correspond with the functional state of the hospital patient and/or the services provided in the hospital (e.g., occupational therapy) thereby resulting in frustration for LTC staff and the family.

#### Low staff ratio (physical opportunity, physical capability)

The respondents indicated that there is a low staff ratio in the homes, resulting in excessive workloads which often overwhelms the care personnel. The high workload of other staff members such as nurses, and support care personnel (Continuing Care Assistants, Physiotherapy and Occupational therapy Assistants) means that they are unable to engage the residents in rehabilitation activities like range of motion and walking as they are overwhelmed by other tasks. In addition to being short-staffed, the physiotherapists and occupation therapists are occupied by other activities such as risk meetings, meetings with the families, and documentation (chart and progress notes) which further reduces the available time to do actual rehabilitation work, resulting in them “picking and choosing” who to see. The low staffing has implications for the residents, who confirmed that only a handful of rehabilitation providers attend to all residents of the home and thus they receive limited care from them. One of the residents illustrated that they expected three visits per week from rehabilitation providers but would count themselves lucky if they got any.

Moreover, there is also a shortage of volunteers who usually offer support services in LTC homes. The volunteers play a range of roles in the homes which can relieve the workload on staff and also improve residents’ mood and participation in rehabilitation activities. The outbreak of the COVID-19 pandemic resulted in the reduction of the number of people volunteering services in the homes. This trend has persisted even after the relaxation of the restrictions imposed during the pandemic. Despite the relaxation of restrictions imposed during the pandemic, volunteers continue to stay away from the LTC homes.

#### Location of the LTC facility (physical opportunity)

The respondents stated that being in rural areas affects the recruitment and retention of rehabilitation providers as they might prefer to work somewhere else. Proximity to stores, and schools could improve functional rehabilitation activities like outdoor walking and community engagement for residents. However, some of the LTC homes are situated far from these resources thereby limiting the scope of possible activities. The importance of location in the ability to engage in walking activities was also confirmed by some of the residents who stated that their current home has more limitations than where they were living previously.

#### Restraint policy (physical and psychological capability, social opportunity)

The prevailing policy in LTC emphasizes risk prevention aiming to prevent residents from sustaining injuries from falls. In some cases, this could mean restraining those who are perceived to be at risk of falling or injuries in wheelchairs and preventing them from engaging in activities. Restraints refer to the limitation of residents' movements or behaviours to minimize the risk of harm to themselves or others [30]. This includes of physical (e.g., belts, bed rails) or medications (sedatives or antipsychotics) to restrict movement and behaviour [30]. In addition to physical restraints, medications are sometimes used to restrain residents who are displaying potentially harmful behaviours from engaging in activities. This is a barrier to rehabilitation as restrained residents are less likely to engage in any activities and might also discourage rehabilitation providers from engaging them in activities like walking. It was also indicated that mobilizing or encouraging such residents to mobilize comes with additional risks as they might be blamed if there is an eventual adverse event like falls or injuries.

### Noisy or busy environment (social and physical opportunity)

Our participants suggested that the nature of the environment affects the mood of the residents and their likelihood to engage in any activity. Residents who like quiet and serene environments might withdraw from all activities when there is noise and activities around them. For instance, some residents stated that they decline participation in group activities because they are not comfortable in noisy or crowded spaces. This was confirmed by staff members who stated that many residents stay in their rooms when holiday activities are going on. Moreover, residents with visual or auditory impairments find it difficult to follow instructions in noisy and busy environments.

### COM-B, intervention functions, and policy categories

Using the Capability, Opportunity, and Motivation (COM-B) model, we present in Table 2 what needs to change to improve rehabilitation for LTC residents living with dementia as well as linked intervention functions and policies to bring about the change. A summary of the plausible intervention functions and policies to stimulate the changes based on the APEASE criteria are presented below.

### Intervention functions

We linked the identified barriers to seven intervention functions: restriction, enablement, training, education, environmental restructuring, modelling, and persuasion. Restriction involves using rules to promote target behaviour by reducing opportunities for engaging in less desired ones. Enablement aims to reduce barriers by enhancing the means to achieve behavioural change, increasing capabilities beyond education and training, and expanding opportunities beyond environmental restructuring. Training improves necessary skills, while education enhances knowledge and understanding of the behaviour. Environmental restructuring modifies physical and social contexts to improve capabilities and opportunities. Modelling creates exemplary behaviour for others to imitate, and persuasion stimulates change by inducing positive or negative feelings [18].

Restriction could be used to tackle the barrier associated with the transition from acute to LTC, rules could be set to improve communication and collaboration between acute, sub-acute, and LTC institutions. Enablement could be used to increase the capability of the staff and providers and overcome the barrier of low staffing through increased recruitment and creating opportunities for more volunteer services. Three barriers; restraint policy, lack of interdisciplinary practice, and communication difficulties were linked to training intervention. Skills training offers opportunities and capabilities to overcome these barriers. Similarly, three barriers including family involvement, shortage of volunteers and communication difficulties were linked to education. With adequate education about dementia management and the potential contributions of the public in care delivery, family members and community dwellers could be offered the opportunity to volunteer their services. Moreover, increasing staff and providers’ knowledge about responsive behaviours and effective communication strategies can improve their capability to provide care for residents.

Restructuring the physical environment of the LTC homes through the creation and procurement of spaces and equipment for exercise can improve the opportunities for more residents to engage in rehabilitation activities. Communication difficulties were also linked to modelling, demonstrating activities might improve residents' understanding of the instructions and their capability to execute such activities. Finally, persuasion was linked to a lack of motivation to engage in activities. The motivation of residents to engage in activities might be improved by adopting a person-centred approach. This might include identifying their personal preferences, sources of motivation, and their limitations.

### Policy categories

In the final level of analysis, we considered policies that will support the delivery of the interventions described in the previous section. A total of five policy categories were identified and they included regulations, guidelines, communication/marketing, environmental/social planning, and fiscal measures. These policies represent decisions that policymakers can use to implement the interventions [18]. Guidelines are policy documents that recommend or mandate practice while regulations are laid down rules and principles to guide behaviour and practice. These could be used to set practice standards and minimum training requirements to competently provide direct care to residents. Communication/marketing involves mass media campaigns to educate and inform stakeholders about the need for change and the means of achieving it. This could include public awareness campaigns targeting the providers, the family, and the community, reinforcing the integral roles they play in the provision of rehabilitation for residents with dementia in LTC. Environmental/social planning refers to the control and design of the physical and social environment to improve the capability and opportunity for care provision while fiscal measures on the other hand involve the use of public tax systems to control the financial costs of practice or behaviour. These two are related as fiscal measures could be used to improve the physical and social context thereby improving the opportunities and capabilities to provide competent and quality care.

## Discussion

This study highlights the barriers to rehabilitation for LTC residents with dementia and identifies practical interventions and policies for overcoming these obstacles. Some of these factors are related to the residents, rehabilitation providers and staff, and the working environment and resources. Our analysis showed that enhancing the capabilities, opportunities, and motivations of all actors in LTC homes can potentially minimize the barriers. Key improvements include better communication with residents, reducing the use of restraints, interdisciplinary practice and making activity spaces and equipment more accessible. These improvements can be achieved through interventions such as training, persuasion, and education. Additionally, implementing policy measures like regulations and guidelines, communications and marketing, environmental/social planning and fiscal measures will help ensure these interventions are effectively carried out.

One of our main findings is that some barriers to rehabilitation are at the intrapersonal level among rehabilitation providers. Dementia presents unique symptoms that pose additional challenges to healthcare providers, consequently affecting their ability to provide competent care [31, 32]. Responsive behaviours were one of the challenges highlighted by our participants as a barrier to providing quality care. Participants reported that both restraints were commonly applied to limit residents’ engagement when they displayed responsive behaviours like agitation. Möhler et al., [30] showed that the current best practice is to encourage the least‐restraint policy. In Canada, various provinces have regulations to minimize the use of restraints in long-term care homes [2, 33]. We identified continued education and training of staff as the most effective intervention for the implementation of the least restrain policy. Our participants identified the GPA training as one of the effective programs for this purpose. The GPA has been shown to improve dementia care self-efficacy, competence, and knowledge among the staff of in-patient medical units [29]. Policy regulation could be used to set such training as a requirement for all providers with direct access to residents with dementia.

Another barrier that could be managed at the intrapersonal and interpersonal levels was the apparent lack of motivation of the residents to engage in meaningful activities. The effectiveness of most rehabilitation activities requires the active participation of the residents which is diminished when they lack the enthusiasm to engage [34]. Our participants reported motivation could be enhanced through several strategies including person-centred care and involving the family member or other familiar faces in the care. This was expected as a previous systematic review on the effectiveness of person-centred care on people with dementia showed that it improves agitation, mood, and quality of life with individualized activities providing the most benefits [35]. Moreover, the adoption of meaningful and personalized functional activities like baking and walking for a purpose (e.g., walking to the dinner table) might achieve higher participation instead of more structured exercises like handgrip or sit-to-stand.

At the policy/environmental level, we identified limited resources as a major constraint to providing rehabilitation. These include equipment needed for rehabilitation activities as well as a shortage of staff. Our participants indicated that the number of rehabilitation staff working in the LTC homes could not meet the current care demand resulting in lower quantity and quality of care provision. This was consistent with the findings of previous studies [34, 36]. Benjamin et al., [36] identified the prohibitive staffing cost of implementing exercise programs as a major barrier where an average exercise program required additional 2.5 support staff and more supervision time. The review also reported that physical activities were not promoted because the staff had excessive work demands [36]. Similarly, while our participants recognized the importance of interdisciplinary practice in care delivery, time constraints and heavy workloads hindered their adoption thereby further limiting the quality of care provided. The allocation of more resources to LTC to improve the human workforce, and care facilities will improve care provision for residents living with dementia.

The utilization of services from community volunteers is another potential means of relieving the work demand. However, volunteer activities in the LTC homes were disrupted by COVID-19-induced restrictions. Our participants reported that despite the relaxation of most of these restrictions, LTC homes continue to record low visits by volunteers. These volunteers perform a series of activities which include providing physical and emotional support for residents and direct support for staff including rehabilitation providers [37]. Before COVID, medium-sized homes regularly received services from 50-100 volunteers who were supported by a few staff members. Presently, LTC homes can no longer count on these services, therefore exacerbating staffing pressure [38]. This trend could be reversed through mass media campaigns to reorient the public on the importance of their services in the care delivery for residents.

Finally, the process of transitioning from an acute care setting (e.g., hospital) to LTC can pose some hindrances to the provision of rehabilitation for the residents. Our participants reported that this transition often results in disruptions in care due to extended waiting times as well as suboptimal communication between the hospitals and LTC. This is challenging for residents and caregivers as there are possibilities of a significant decline in function during the waiting period often leading to readmission into acute care within 30 days [39]. Moreover, our participants stated that it also creates stressful conditions between LTC staff and the families especially where the residents are not able to regain or maintain the level of functioning they had before admission to the homes. To mitigate this challenge, our analysis suggests that rules and regulations could be used to strengthen communication and collaboration among institutions. This aligns with previous recommendations which advocated for increased collaboration between hospitals and post acute care institutions [40–42].

The findings of this study have significant implications for the practice and policy in LTC settings. Enhancing the capabilities of rehabilitation providers through targeted training and education can improve care quality and reduce the reliance on restraints. Additionally, fostering motivation among residents through personalized and meaningful activities can increase their participation in rehabilitation. At the policy level, allocating more resources to LTC, improving transitions from acute care, and promoting volunteer involvement can address systemic barriers to rehabilitation.

While this study provides valuable insights into the barriers and facilitators of rehabilitation for LTC residents with dementia, several limitations should be acknowledged. First, the study was conducted across 17 privately owned LTC homes in Nova Scotia, Canada with a relatively small number of participants in each group. This limited sample size may not be representative of all LTC homes or of diverse geographical locations, which restricts the generalizability of the findings to other settings or populations. Second, we relied on LTC staff to identify and suggest potential participants, which might have introduced selection bias. There is a possibility that those who were more motivated or had more favourable views about rehabilitation were more likely to participate, potentially skewing the findings. Finally, we relied heavily on self-reported data, which can be subject to social desirability bias. Our participants might have provided responses they perceived as socially acceptable or favorable, rather than their true experiences or opinions.

## Conclusion

Despite the above limitations, we highlighted the multifaceted barriers to rehabilitation for LTC residents with dementia and proposed practical interventions and policies to overcome these challenges. Enhancing communication, reducing restraints, promoting interdisciplinary practice, and addressing resource limitations can significantly improve the quality of care. Future efforts should focus on implementing these interventions and evaluating their long-term impact to ensure sustained benefits for residents with dementia in LTC settings. Specifically, future research should explore the long-term effects of personalized rehabilitation activities on health outcomes for residents with dementia. Studies could also investigate the impact of interdisciplinary practices in LTC settings and how policy changes, such as increased funding and better staff-to-resident ratios, influence rehabilitation effectiveness. Further research is needed to understand the role of community volunteers post-pandemic and strategies to reintegrate them into LTC settings effectively.

## Supplementary Information

Supplementary Material 1.

Supplementary Material 2.

## Data Availability

The datasets used and analyzed during the current study are available from the corresponding author on reasonable request.

## Abbreviations

LTC: Long-term care
BCW: Behavioral Change Wheel
APEASE: Acceptability, Practicability, Effectiveness, Affordability, Side-effects, and Equity
ADL: Activities of Daily Living
COM-B: Capability, Opportunity, and Motivation behaviour model (COM-B)
SEM: Socioecological Model

## References

[1] Alzheimer Society of Canada. Alzheimer Society of Canada. 2024. Dementia numbers in Canada. Available from: http://alzheimer.ca/en/about-dementia/what-dementia/dementia-numbers-canada. Cited 2024 Feb 25.

[2] Sandra Lopes. Understanding long-term care homes. 2023. Available from: https://www.ola.org/sites/default/files/node-files/llrs/document/pdf/2023/2023-06/Understanding%20Long%20Term%20Care%20Homes.pdf#:~:text=URL%3A%20https%3A%2F%2Fwww.ola.org%2Fsites%2Fdefault%2Ffiles%2Fnode. Cited 2024 Oct 6.

[3] Canadian Institute for Health Information. Dementia in long-term care. 2023. Available from: https://www.cihi.ca/en/dementia-in-canada/dementia-care-across-the-health-system/dementia-in-long-term-care. Cited 2023 Aug 20.

[4] Bennett S, Laver K, Voigt-Radloff S, Letts L, Clemson L, Graff M, et al. Occupational therapy for people with dementia and their family carers provided at home: a systematic review and meta-analysis. BMJ Open. 2019;9(11):e026308. 31719067 10.1136/bmjopen-2018-026308 PMC6858232

[5] Helvik AS, Selbæk G, Benth JŠ, Røen I, Bergh S. The course of neuropsychiatric symptoms in nursing home residents from admission to 30-month follow-up. PLoS One. 2018 Oct 18;13(10). Available from: http://ezproxy.library.dal.ca/login?url=https://search.ebscohost.com/login.aspx?direct=true&db=psyh&AN=2018-53020-001&site=ehost-live. 10.1371/journal.pone.0206147 PMC6193723 30335840

[6] Lane NE, Wodchis WP, Boyd CM, Stukel TA. Disability in long-term care residents explained by prevalent geriatric syndromes, not long-term care home characteristics: a cross-sectional study. BMC Geriatr. 2017;10(17):49. 10.1186/s12877-017-0444-1 PMC5301427 28183274

[7] Thomas DW. A case study on the effects of a retrofitted dementia special care unit on resident behaviors. Am J Alzheimers Dis. 1996;1996:8–14.

[8] Hirdes JP, Mitchell L, Maxwell CJ, White N. Beyond the “iron lungs of gerontology”: using evidence to shape the future of nursing homes in Canada. Can J Aging. 2011;30(3):371–90. 21851753 10.1017/S0714980811000304

[9] Nguyen TV, Dang HT, Burns MJ, Dao HH, Nguyen TN. Impairment in activities of daily living and readmission in older patients with heart failure: a cohort study. BMJ Open. 2021;11(2):e044416. 33619200 10.1136/bmjopen-2020-044416 PMC7903094

[10] Edemekong PF, Bomgaars DL, Sukumaran S, Schoo C. Activities of Daily Living. In: StatPearls. Treasure Island (FL): StatPearls Publishing; 2024. Available from: http://www.ncbi.nlm.nih.gov/books/NBK470404/. Cited 2024 Feb 27. 29261878

[11] Clare L. Rehabilitation for people living with dementia: a practical framework of positive support. PLoS Med. 2017;14(3):e1002245. 28267744 10.1371/journal.pmed.1002245 PMC5340348

[12] Chu CH, Puts M, Brooks D, Parry M, McGilton KS. A feasibility study of a multifaceted walking intervention to maintain the functional mobility, activities of daily living, and quality of life of nursing home residents with dementia. Rehabil Nurs. 2020;45(4):204–17. 30325875 10.1097/rnj.0000000000000186

[13] McArthur C, Hirdes J, Berg K, Giangregorio L. Who receives rehabilitation in canadian long-term care facilities? A cross-sectional study. Physiother Can. 2015;67(2):113–21. 25931661 10.3138/ptc.2014-27 PMC4407121

[14] Buddingh S, Liang J, Allen J, Koziak A, Buckingham J, Beaupre LA. Rehabilitation for long-term care residents following hip fracture: a survey of reported rehabilitation practices and perceived barriers to delivery of care. J Geriatr Phys Ther. 2013;36(1):39–46. 22576242 10.1519/JPT.0b013e3182569b4f

[15] Foley T, Sheehan C, Jennings AA, O’Sullivan T. A qualitative study of the dementia-care experiences and educational needs of physiotherapists in the Republic of Ireland. Physiotherapy. 2020;1(107):267–74. 10.1016/j.physio.2019.08.006 32026828

[16] Ries JD. Rehabilitation for individuals with dementia: facilitating success. Curr Geri Rep. 2018;7(1):59–70.

[17] Hall AJ, Manning F, Goodwin V. Qualitative study exploring health care professionals’ perceptions of providing rehabilitation for people with advanced dementia. BMJ Open. 2023;13(7):e072432. 37524545 10.1136/bmjopen-2023-072432 PMC10391829

[18] Michie S, van Stralen MM, West R. The behaviour change wheel: a new method for characterising and designing behaviour change interventions. Implement Sci. 2011;6(1):42. 21513547 10.1186/1748-5908-6-42 PMC3096582

[19] Lun P, Gao J, Tang B, Yu CC, Jabbar KA, Low JA, et al. A social ecological approach to identify the barriers and facilitators to COVID-19 vaccination acceptance: a scoping review. PLoS One. 2022;17(10):e0272642. 36191018 10.1371/journal.pone.0272642 PMC9529136

[20] Tanwir F, Moideen S, Habib R. Interviews in healthcare: a phenomenological approach a qualitative research methodology. JPHI. 2021;4(2):10–5.

[21] Neubauer BE, Witkop CT, Varpio L. How phenomenology can help us learn from the experiences of others. Perspect Med Educ. 2019;8(2):90–7. 30953335 10.1007/s40037-019-0509-2 PMC6468135

[22] Tong A, Sainsbury P, Craig J. Consolidated criteria for reporting qualitative research (COREQ): a 32-item checklist for interviews and focus groups. Int J Qual Health Care. 2007;19(6):349–57. 17872937 10.1093/intqhc/mzm042

[23] Guest G, Bunce A, Johnson L. How many interviews are enough?: An experiment with data saturation and variability. Field Methods. 2006;18(1):59–82.

[24] Otter.ai. Otter.ai - AI Meeting Note Taker & Real-time AI Transcription. 2023. Available from: https://otter.ai/. Cited 2024 Jun 24.

[25] Hsieh HF, Shannon SE. Three approaches to qualitative content analysis. Qual Health Res. 2005;15(9):1277–88. 16204405 10.1177/1049732305276687

[26] Graneheim UH, Lundman B. Qualitative content analysis in nursing research: concepts, procedures and measures to achieve trustworthiness. Nurse Educ Today. 2004;24(2):105–12. 14769454 10.1016/j.nedt.2003.10.001

[27] Neergaard MA, Olesen F, Andersen RS, Sondergaard J. Qualitative description – the poor cousin of health research? BMC Med Res Methodol. 2009;9(1):52. 19607668 10.1186/1471-2288-9-52 PMC2717117

[28] Scarneo SE, Kerr ZY, Kroshus E, Register-Mihalik JK, Hosokawa Y, Stearns RL, et al. The socioecological framework: a multifaceted approach to preventing sport-related deaths in high school sports. J Athl Train. 2019;54(4):356–60. 30870600 10.4085/1062-6050-173-18 PMC6522086

[29] Crandall J, Coatsworth-Puspoky R, Schlegel K, Beker L, McLelland VC, Martin LS. implementing gentle persuasive approaches dementia education for staff on in-patient medicine units: a program evaluation. Dementia (London). 2022;21(4):1173–99. 35081811 10.1177/14713012211070148 PMC9109211

[30] Möhler R, Richter T, Köpke S, Meyer G. Interventions for preventing and reducing the use of physical restraints for older people in all long‐term care settings. Cochrane Database of Systematic Reviews. 2023;(7). Available from: https://www.cochranelibrary.com/cdsr/doi/10.1002/14651858.CD007546.pub3/full. Cited 2024 May 28. 10.1002/14651858.CD007546.pub3 PMC10374410 37500094

[31] Alzheimer's Association. 2022 Alzheimer's disease facts and figures. 2022. Available from: 10.1002/alz.12638. 10.1002/alz.12638 35289055

[32] Wimo A, Ali GC, Guerchet M, Prince M, Prina M, Wu YT. World Alzheimer Report 2015: The global impact of dementia: An analysis of prevalence, incidence, cost and trends. 2015. Available from: https://www.alzint.org/resource/world-alzheimer-report-2015/. Cited 2024 May 28.

[33] CIHI. Restraint Use in Long-Term Care. 2023. Available from: https://www.cihi.ca/en/indicators/restraint-use-in-long-term-care. Cited 2024 Oct 6.

[34] Shirozhan S, Arsalani N, SeyedBagherMaddah S, Mohammadi-Shahboulaghi F. Barriers and facilitators of rehabilitation nursing care for patients with disability in the rehabilitation hospital: a qualitative study. Front Public Health. 2022;10:931287. 36033757 10.3389/fpubh.2022.931287 PMC9402936

[35] Kim SK, Park M. Effectiveness of person-centered care on people with dementia: a systematic review and meta-analysis. Clin Interv Aging. 2017;17(12):381–97. 10.2147/CIA.S117637 PMC5322939 28255234

[36] Benjamin K, Edwards N, Ploeg J, Legault F. Barriers to physical activity and restorative care for residents in long-term care: a review of the literature. J Aging Phys Act. 2014;22(1):154–65. 23434919 10.1123/japa.2012-0139

[37] Steunenberg B, van der Mast R, Strijbos MJ, Inouye SK, Schuurmans MJ. How trained volunteers can improve the quality of hospital care for older patients. A qualitative evaluation within the Hospital Elder Life Program (HELP). Geriatr Nurs. 2016;37(6):458–63. 27471215 10.1016/j.gerinurse.2016.06.014

[38] Ramesar V. Absence of volunteers creates staffing pressures at N.S. nursing homes. CBC News. 2020. Available from: https://www.cbc.ca/news/canada/nova-scotia/nursing-homes-pandemic-recreation-covid-19-1.5692866. Cited 2024 Mar 15.

[39] Britton MC, Ouellet GM, Minges KE, Gawel M, Hodshon B, Chaudhry SI. Care transitions between hospitals and skilled nursing facilities: perspectives of sending and receiving providers. Jt Comm J Qual Patient Saf. 2017;43(11):565–72. 29056176 10.1016/j.jcjq.2017.06.004 PMC5693352

[40] Berkowitz RE, Fang Z, Helfand BKI, Jones RN, Schreiber R, Paasche-Orlow MK. Project ReEngineered Discharge (RED) lowers hospital readmissions of patients discharged from a skilled nursing facility. J Am Med Dir Assoc. 2013;14(10):736–40. 23608528 10.1016/j.jamda.2013.03.004

[41] Sandvik D, Bade P, Dunham A, Hendrickson S. A hospital-to-nursing home transfer process associated with low hospital readmission rates while targeting quality of care, patient safety, and convenience: a 20-year perspective. J Am Med Dir Assoc. 2013;14(5):367–74. 23375522 10.1016/j.jamda.2012.12.007

[42] Yoo JW, Jabeen S, Bajwa T, Kim SJ, Leander D, Hasan L, et al. Hospital readmission of skilled nursing facility residents: a systematic review. Res Gerontol Nurs. 2015;8(3):148–56. 25710452 10.3928/19404921-20150129-01

