# Supplementary material for: Barriers and facilitators to providing rehabilitation for long-term care residents with dementia: a qualitative study
Source: BMC Geriatr. 2024 Oct 15;24:838. doi: 10.1186/s12877-024-05433-z (PMC11476797; doi:10.1186/s12877-024-05433-z)
Supplement: Supplementary file 1 — Supplementary Material 1. [file 12877_2024_5433_MOESM1_ESM.docx]

**Supplementary Table 1:**

Consolidated criteria for reporting qualitative studies (COREQ): 32-item checklist

| **Items** | **Page #** |
| --- | --- |
| **Domain 1: Research team and reflexivity** | |
| *Personal Characteristics* | |
| Interviewer/facilitator - Which author/s conducted the interview or focus group? | 8 |
| Credentials - What were the researcher's credentials? | 10 |
| Occupation - What was their occupation at the time of the study? | 10 |
| Gender - Was the researcher male or female? | 10 |
| Experience and training - What experience or training did the researcher have? | 10 |
| *Relationship with participants* | |
| Relationship established - Was a relationship established prior to study commencement? | 7 |
| Participant knowledge of the interviewer - What did the participants know about the researcher? | 7 |
| Interviewer characteristics - What characteristics were reported about the interviewer/facilitator? | 10 |
| **Domain 2: study design** | |
| *Theoretical framework* | |
| Methodological orientation and Theory - What methodological orientation was stated to underpin the study? | 6 |
| *Participant selection* | |
| Sampling - How were participants selected? | 7 |
| Method of approach - How were participants approached? | 7 |
| Sample size - How many participants were in the study? | 7, 11 |
| Non-participation - How many people refused to participate or dropped out? Reasons? | 8 |
| *Setting* | |
| Setting of data collection - Where was the data collected? | 8 |
| Presence of non-participants - Was anyone else present besides the participants and researchers? | 8 |
| Description of sample - What are the important characteristics of the sample? | 6-7 |
| *Data collection* | |
| Interview guide - Were questions, prompts, guides provided by the authors? Was it pilot tested? | 8 |
| Repeat interviews - Were repeat interviews carried out? If yes, how many? | n/a |
| Audio/visual recording - Did the research use audio or visual recording to collect the data? | 8 |
| Field notes - Were field notes made during and/or after the interview or focus group? | 8 |
| Duration - What was the duration of the interviews or focus group? | 8 |
| Data saturation - Was data saturation discussed? | 7 |
| Transcripts returned - Were transcripts returned to participants for comment and/or correction? | n/a |
| **Domain 3: analysis and findings** | |
| *Data analysis* | |
| Number of data coders - How many data coders coded the data? | 8 |
| Description of the coding tree - Did authors provide a description of the coding tree? | (see Table 1) |
| Derivation of themes - Were themes identified in advance or derived from the data? | 8 |
| Software - What software, if applicable, was used to manage the data? | 8 |
| Participant checking - Did participants provide feedback on the findings? | n/a |
| *Reporting* | |
| Quotations presented - Were participant quotations presented to illustrate the themes / findings? Was each quotation identified? | (see Table 1) |
| Data and findings consistent - Was there consistency between the data presented and the findings? | 11-21 |
| Clarity of major themes - Were major themes clearly presented in the findings? | 11-18 |
| Clarity of minor themes - Is there a description of diverse cases or discussion of minor themes? | 11-18 |
