# Supplementary material for: Barriers and facilitators to providing rehabilitation for long-term care residents with dementia: a qualitative study
Source: BMC Geriatr. 2024 Oct 15;24:838. doi: 10.1186/s12877-024-05433-z (PMC11476797; doi:10.1186/s12877-024-05433-z)
Supplement: Supplementary file 2 — Supplementary Material 2. [file 12877_2024_5433_MOESM2_ESM.docx]

**Semi-structured Interview 1 Guide**

**Pre-amble**

I want to ask you what makes it harder or easier for you/your family member/the resident to participate in rehabilitation/exercise. This information will help us develop a rehabilitation program that is resident-centred. I will be audio-recording the conversation. If you have something to say that you do not want audio-recorded you can tell me and I will turn off the audio-recorder. You can also tell me afterwards if there is something you want removed. Your responses will be kept confidential and you will not be identified in any presentations or publications of the results.

**1. What makes it or would make it hard for you/your family member/the resident to participate in rehabilitation or exercise?**

*Follow-up/probing questions:*

a) (For each item identified) Why does it make it hard?

b) What else makes it hard?

c) Why would you/your family member/the resident not participate in rehabilitation or exercise?

d) What would you like rehabilitation professionals to know about rehabilitation for people with dementia?

**2. What makes it easier or would make it easier for you/your family member/the resident to participate in rehabilitation or exercise?**

*Follow-up/probing questions:*

a) (For each item identified) Why does it make it easier?

b) What else makes it easier?

c) Why would you/your family member/the resident participate in rehabilitation or exercise?

**Semi-structured Interview 2 Guide**

**Pre-amble**

I want to show what we learned from you last time and see if you agree/disagree with what we found, and if there is anything else you would like to add. This information will help us develop a rehabilitation program that is resident-centred. I will be audio-recording the conversation. If you have something to say that you do not want audio-recorded you can tell me and I will turn off the audio-recorder. You can also tell me afterwards if there is something you want removed. Your responses will be kept confidential and you will not be identified in any presentations or publications of the results.

**PRESENT PRELIMINARY RESULTS**

**1. Do you agree or disagree with (result)?**

*Follow-up/probing questions:*

a) Why or why not?

b) What would you like to add? What else do you want us to know? What else do you want rehabilitation professionals to know?
